# Introduction: Proceedings of the First and Second European Workshops on Preterm Labour of the Special Non-Invasive Advances in Fetal and Neonatal Evaluation (SAFE) Network of Excellence

**DOI:** 10.1186/1471-2393-7-S1-S1

**Published:** 2007-06-01

**Authors:** Jörg Strutwolf

**Affiliations:** 1 Nanobiotechnology and Bioanalysis Group, Department of Chemical Engineering, Universitat Rovira I Virgili, Avd. Països Catalans 26, 43007 Tarragona, Spain

This special issue of *BMC Pregnancy and Childbirth *comprises the proceedings of papers presented at the 1^st ^and 2^nd ^European SAFE Workshops on Preterm Labour, held on 22 June 2005 and 21–22 September 2006 in Tarragona, Spain. These workshops were sponsored by the EU funded SAFE Network of Excellence , as part of the activities of Workpackage 4 'New Technologies for Improved Non-Invasive Prenatal Diagnosis and Enhanced Neonatal Screening'.

SAFE, the Special Non-Invasive Advances in Fetal and Neonatal Evaluation Network, is a European Union-funded Network of Excellence (LSHB-CT-2004-503243) that started on 1 March 2004, and is set to run for five years. It is sponsored under the EU's Framework programme 6 'Life Sciences, Genomics and Biotechnology for Health'. The SAFE Network aims to implement routine non-invasive prenatal diagnosis and cost-effective screening of newborns through the creation of long-term partnerships between paediatricians, obstetricians, molecular biologists, and clinical geneticists as well as leading thinkers in social sciences and ethics worldwide. SAFE brings together leading experts from these key disciplines in a programme designed to enhance the clinical usefulness of Non Invasive Prenatal Diagnosis (NIPD) and Neonatal Screening (NS) within and beyond the European Community for the benefit of families worldwide. If you would like more information about this project please email safeproject@warwick.ac.uk or refer to the website, .

The papers presented in this collection provide an overview of the current state of research in the field of preterm labour in Europe, covering diverse areas such as mechanisms and mediators, methods of prediction and prevention, and new drug development. During the course of the workshops it became more and more clear that understanding, detecting and preventing a highly complex issue such as preterm labour requires a multi-disciplinary approach and a strong interaction between different research groups. We hope that the workshops and this collection of papers will contribute towards a better understanding of preterm labour. The workshops in Tarragona have provided a very useful forum for a core group of European investigators from academia and industry to meet in a relaxed atmosphere and discuss the most recent clinical, laboratory and epidemiological findings in preterm birth and related pregnancy and neonatal complications. The workshops also provided a good discussion platform for new approaches to the diagnosis and management of preterm labour. This is reflected in the range of manuscripts presented in this issue of *BMC Pregnancy and Childbirth*.

We would like to thank Ferring Pharmaceuticals , PerkinElmer  and Merck-Serono  for covering the publication costs.

Organizing committee: Ciara K O'Sullivan and Jörg Strutwolf

Scientific advisory board: Andrés López Bernal, Guy Germain and Michael Taggart

